# Triticale Bran Alkylresorcinols Enhance Resistance to Oxidative Stress in Mice Fed a High-Fat Diet

**DOI:** 10.3390/foods5010005

**Published:** 2016-01-05

**Authors:** Rania Agil, Zachary R. Patterson, Harry Mackay, Alfonso Abizaid, Farah Hosseinian

**Affiliations:** 1 Food Science & Nutrition Program, Department of Chemistry, Carleton University, Ottawa, ON K1S 5B6, Canada; Rania_agil@carleton.ca; 2 Department of Neuroscience, Carleton University, Ottawa, ON K1S 5B6, Canada; zack.patterson@gmail.com (Z.R.P.); harry.m@gmail.com (H.M.); Alfonso.AbizaidBucio@carleton.ca (A.A.); 3 Institute of Biochemistry, Carleton University, Ottawa, ON K1S 5B6, Canada

**Keywords:** *Triticosecale*, triticale bran, alkylresorcinols, body composition, glucose tolerance, antioxidant activity, oxidative stress, magnetic resonance imaging (MRI), obesity

## Abstract

Triticale (× *Triticosecale* Whitm.) is a cereal grain with high levels of alkyresorcinols (AR) concentrated in the bran. These phenolic lipids have been shown to reduce or inhibit triglyceride accumulation and protect against oxidation; however, their biological effects have yet to be evaluated *in vivo*. The purpose of this study was to determine the effects of ARs extracted from triticale bran (TB) added to a high–fat diet on the development of obesity and oxidative stress. CF-1 mice were fed a standard low-fat (LF) diet, a 60% high-fat diet (HF) and HF diets containing either 0.5% AR extract (HF-AR), 10% TB (HF-TB), or 0.5% vitamin E (HF-VE). Energy intake, weight gain, glucose tolerance, fasting blood glucose (FBG) levels, and body composition were determined. Oxygen radical absorbance capacity (ORAC), superoxide dismutase (SOD) activity, and glutathione (GSH) assays were performed on mice liver and heart tissues. The findings suggest that ARs may serve as a preventative measure against risks of oxidative damage associated with high-fat diets and obesity through their application as functional foods and neutraceuticals. Future studies aim to identify the *in vivo* mechanisms of action of ARs and the individual homologs involved in their favorable biological effects.

## 1. Introduction

The rising incidence rate of obesity has become a serious public health concern, resulting in a growing consumer demand for a healthy diet by means of natural, health-promoting products [1,2]. Obesity is a disorder associated with an increased risk of numerous diseases including type II diabetes and cardiovascular disease (CVD). Studies suggest that the co-morbidities in obesity are a result of oxidative stress as a chief underlying source [3]. Oxidative stress is an imbalance in reactive oxygen species (ROS) production that overwhelms the body’s antioxidant defense system. This defense system includes both enzymatic and nonenzymatic mechanisms to neutralize and detoxify ROS and reactive intermediates [4,5]. Some of the major contributors to this system are superoxide dismutase (SOD), which catalyzes the conversion of highly reactive superoxide (O_2_^−^) molecules into a less reactive product, hydrogen peroxide (H_2_O_2_). Enzymes glutathione peroxidase (GPx) and catalase can further neutralize H_2_O_2_ by decomposition into water and oxygen. Antioxidant enzymes rely on endogenous antioxidants such as glutathione (GSH) for the hydrogen and electron donations necessary for the reduction and detoxification of ROS [5]. Fat accumulation correlates with oxidative stress, as evidenced by increases in ROS production, the ratio of oxidized glutathione to reduced glutathione (GSSG/GSH), and NADPH oxidase expression, as well as a decrease in antioxidant enzyme expression in the adipose tissue of obese mice [6].

Whole grain consumption is associated with a variety of health benefits including a reduced risk of obesity and related diseases. Whole grains are rich in nutrients and phytochemicals, which have been linked to the improvements seen in terms ofantioxidant protection, weight loss, BMI, and blood-lipid metabolism in subjects fed a diet rich in whole grains [7,8,9]. The health-related functionality of whole grains is principally due to the presence of bioactive compounds in the bran. Some of these health benefits may be attributed to alkylresorcinols (ARs), bioactive compounds present almost exclusively in the bran. These phenolic lipids possess a polar aromatic ring and a hydrophobic alkyl chain, generally at the C5 position. Hydroxyl groups are attached to the aromatic ring at C1 & C3 and may serve as sources of ARs’ radical scavenging activity through hydrogen donation [10]. Due to similarities in structural properties, ARs are often compared to vitamin E (tocopherols), which is a well-established antioxidant [11], and studies have reported similarities in their metabolism after ingestion [10,12,13]. ARs have also been shown to reduce or inhibit triglyceride accumulation [14], in addition to their anti-inflammatory properties [15] and antioxidant protection [16]. In a recent study by Oishi *et al.*, mice fed a 28% fat and 20% sucrose diet showed positive physiological effects when their diet was also supplemented with ARs (0.4% wheat bran ARs) in comparison to mice fed the same diet lacking ARs [17]. Researchers found that the incorporation of ARs in the diet suppressed the risks of obesity and glucose intolerance generally associated with a high fat, high sucrose diet by increasing insulin sensitivity and cholesterol excretion. This is one of very few studies that have investigated the physiological effects of ARs. Nonetheless, the antioxidant potential of ARs *in vivo* is a question that remains inconclusive and has yet to be evaluated. Thus, the objective of this study was to determine the effects of AR extracts from triticale bran (TB) added to a high–fat diet on the development of obesity and oxidative stress.

## 2. Materials and Methods

### 2.1. Chemicals and Reagents

Acetone was purchased from Caledon Laboratories Ltd. (Georgetown, ON, Canada). Alkylresorcinol standards were ordered from ReseaChem GmbH (Burgdorf, Switzerland). Potassium phosphate buffer (KPi) was purchased from Mallinckrodt (Paris, KY, USA). Fluorescein, Trolox, 2,2′-azobis(2-methylpropionamidine)dihydrochloride (AAPH), super oxide dismutase, manganese(II) chloride (MnCl_2_), 5,5′-dithiobis-(2-nitrobenzoic acid) (DTNB), glutathione reductase (GR), and 2-vinylpyridine (VP) were purchased from Sigma-Aldrich (Oakville, ON, Canada). Ethylenediaminetetraacetic acid (EDTA), sulfosalicylic acid (SA), glutathione (GSH), bovine serum albumin (BSA), nicotinamide adenine dinucleotide (NADH), 2-mercaptoethanol (MeSH), and nicotinamide adenine dinucleotide phosphate (NADPH) were purchased from BioShop (Burlington, ON, Canada). Oxidized glutathione (GSSG) was purchased from Santa Cruz Biotechnology (Dallas, TX, USA).

### 2.2. Sample Preparation and Analysis

Triticale bran (TB) was provided by Agriculture and Agri-Food Canada (Lethbridge, Alberta). Bran was milled to a 2 mm particle size prior to extraction using a Thomas Wiley Mill (model ED-5, Arthur H. Thomas Co., Philadelphia, PA, USA). Complete methods of extraction, characterization, and quantification of ARs were followed in accordance with our previous studies [18]. Alkylresorcinols were extracted from TB for 24 h with acetone, at a 1:40 solid-to-solvent ratio (*w*/*v*; g/mL). The extracted solution had all acetone removed by rotary evaporation using a BüchiRotavapor R-215 (New Castle, DE, USA). The dried product was weighed and stored at −20 °C prior to use.

An Alliance HPLC system e2695 Separation Module with a photodiode array detector from Waters (Milford, MA, USA) equipped with a reverse-phase C18 column (150 × 4.6 mm, 5 μm) and Empower 3 software was used for analysis. AR sample extracts and standards (C15:0, C17:0, C19:0, C21:0, C23:0, and C25:0) were prepared in methanol and concentrations were determined according to the calibration curve (0.125 to 2 mg/mL) of each standard. The method of HPLC analysis was modified from Gunenc *et al.* [19] to separate and quantify the total AR content and homolog composition present in our extract. A gradient system using 4% acetic acid in water (Solvent A) and 1% acetic acid in methanol (Solvent B) was applied at a flow rate of 1 mL/min, with a column temperature of 35 °C and an injection volume of 10 μL. The gradient system used initiated at 90% B for 10 min, followed by 100% B for 30 min, and returned to 90% B for a final 10 min.

### 2.3. Animals and Diets

The study protocol was approved by the Local Ethics Committee at Carleton University and all experimental procedures complied with the Canadian Council on Animal Care (CCAC) guidelines. Male CF-1 mice (~six weeks old; *n* = 40) were obtained from Charles River Laboratories (St. Constant, QC, Canada). Throughout the entirety of the study, mice were maintained on a standard 12 h light dark cycle (lights on at 08:00). Mice were allowed to acclimatize to vivarium conditions for one week, during which time they were left undisturbed with *ad libitum* access to water supply and conventional chow (2014 Teklad Global 14% Protein Rodent Maintenance Diet, Harlan Laboratories, Mississauga, ON, Canada). Following this habituation period, a two-week baseline period was carried out in which mice continued to be fed the same conventional chow with their food intake and body weight measures recorded daily. Subsequently, animals were randomly assigned to one of five groups (*n* = 8 per group), each of which were fed different diets for a period of 10 experimental weeks. Groups were categorized by diet exposure type as follows: (A) low-fat conventional chow for maintenance; (B) high-fat TD.06414 from Harlan Laboratories; (C) high-fat containing 0.5% Vitamin E (5 g/kg feed); (D) high-fat containing 0.5% AR extract (5 g/kg feed); and (E) high-fat containing 10% TB (100 g/kg feed) with groups designated LF, HF, HF-VE, HF-AR, and HF-TB respectively. The compositions of the control LF and HF diets are shown in Table 1.

**Table 1:** Composition of the control diets.

Ingredient	Low-Fat Diet	High-Fat Diet
Carbohydrate (%)	48.0	27.3
Protein (%)	14.3	23.5
Fat (%)	4.0	34.3
Saturated (%)	0.7	12.7
Monounsaturated (%)	0.8	16.1
Polyunsaturated (%)	2.5	5.5
Fibre (%)	22.1	6.5
Soluble (%)	4.1	−
Insoluble (%)	18.0	6.5
Energy density (kcal/g)	2.9	5.1
Energy from carbohydrate (%)	67.3	21.3
Energy from protein (%)	20.1	18.4
Energy from fat (%)	12.6	60.3

Body weight and food intake were recorded daily. The total duration of the study was 12 weeks and at the end of this period, mice were fasted for 12 h prior to sacrifice by decapitation. Blood glucose levels were measured at time of death using a Contour^®^ next EZ Blood Glucose Monitoring System (Bayer Inc., Toronto, ON, Canada) and organs were stored at −80 °C immediately after being removed.

### 2.4. Glucose Tolerance Test (GTT)

During the experimental period, a glucose tolerance test was carried for all groups by intraperitoneal (IP) injection of 20% d-glucose solution to overnight- fasted animals (12 h fast). Blood samples were collected from the tail vein and glucose levels measured by Contour Blood Glucose Monitor at time 0 (prior to injection), 15, 30, 60, and 120 min after injection to determine the rate of reduction of glucose level. The dose of glucose solution administered was 0.5% (0.5 mL/100 g) of the mouse’s total body weight.

### 2.5. Carcass Analysis

Magnetic resonance imaging (MRI) was carried out courtesy of Health Canada using an EchoMRI4in1TM mouse composition analyzer (EchoMRI, Houston, TX, USA) to measure the % body fat and % lean fat of the decapitated animal carcasses.

### 2.6. Tissue Preparation

Dissected liver and heart tissues were fixed in solution at a ratio of 1:5 (*w*/*v*; g/mL), homogenized using a Tissuemiser 130 V 50/60 HZ, 125 W (Fisher Scientific, Ottawa, ON, Canada), centrifuged at 12,000 g for 15 min at 4 °C, and the supernatant was collected and stored at −80 °C until analysis ensued. Tissue samples used for GSH assays were homogenized in ice-cold 5% SA that had been briefly bubbled in nitrogen gas. Samples used for SOD and ORAC assays were homogenized in a solution of 100 mM potassium phosphate buffer solution (pH 7.5) with 1 mM EDTA.

### 2.7. Protein Determination

Protein levels were determined based on the method proposed by Bradford M.M. [20], using bovine serum albumin as a standard. Absorbance was read at 595 nm using a SpectraMax 340PC384 microplate reader (Molecular Devices, Sunnyvale, CA, USA).

### 2.8. Antioxidant Activity Assays

#### 2.8.1. Oxygen Radical Absorbance Capacity (ORAC) Assay

ORAC assays were carried out with minor modifications of procedures previously described by us [18]. All solutions were prepared in 100 mM potassium phosphate buffer (pH 7.5) which was also used as the blank. Varying concentrations of Trolox (a water-soluble vitamin E analog) were used as standard to create a reference range. Fluorescence was measured using an automated plate reader (FLx800 with Gen5 software, BioTek Instruments, Winooski, VT, USA) for a total runtime of 90 min, at excitation and emission wavelengths of 485 and 525 nm. Final results were calculated as Trolox equivalents per mg of protein.

#### 2.8.2. Super Oxide Dismutase (SOD) Activity

The activity of SOD in tissue samples was measured indirectly by monitoring the inhibition of superoxide-induced NADH oxidation, seen as a decrease in absorbance at 340 nm over a period of 10 min [21]. The following solutions (10 μL each) were added sequentially to a 96-well plate: 50 mM EDTA, 25 mM MnCl2 , 2.7 mM NADH, and 50 μL of supernatant (6–8 different concentrations), blank (100 mM KPi), or standard (1 μg/mL SOD). The reaction was initiated by the final addition of 39 mM MeSH. One unit of SOD activity represents the amount of enzyme in the sample that inhibits NADH oxidation reaction by 50%. Results are expressed in U of SOD/mg of protein.

#### 2.8.3. Gluatathione (GSH) Assay

The assay for glutathione followed procedures similar to those previously described by Griffith O.W. [22]. It is based on the reaction of GSH with DTNB to produce a yellow end-product that absorbs at 412 nm. Thus the concentration of GSH in the sample is dependent on the rate of TNB production. To measure total GSH, the following solutions were sequentially added to a 96-well plate: 0.55 mM NADPH (100 µL), 1.32 mM DTNB (10 µL), sample, standard or blank (10 µL), and 11 U/mL GR (10 µL). Ice-cold 5% SA was used as a blank and to prepare GSH standard solutions (1.25, 2.5, 5, 10, and 20 μM). To determine the amount of GSSG within the samples, samples (50 µL) and a stock solution of GSSG standard (50 µL) were individually treated with 60 μL of a 1:90 solution containing VP and 500 mM KPi (*v*/*v*). This process derivatizes GSH in order to quantify GSSG levels exclusive of GSH within the sample. In a separate 96-well plate, pre-treated samples were run against a GSSG standard curve (0.1, 0.2, 0.4, 0.7, 1, 1.5, and 2.0 μM) following the aforementioned procedure for total GSH. In determining total GSH, and GSSG, the actual concentration of GSH can be deduced using the following formula:

Total = GSH + 2GSSG.

### 2.9. Statistical Analysis

Three of the mice, one from group LF (*n* = 7) and two from HF (*n* = 6), died prior to the end of the study period, thus all data obtained from these mice have been excluded from the final results and statistical analyses. All experiments and analyses were performed at least in triplicate. Results are presented as means ± SEM. Data were statistically analyzed by one-way analysis of variance (ANOVA) followed by Duncan’s multiple range test (DMRT). Means were considered significantly different at *p* < 0.05.

## 3. Results

### 3.1. Characterization of ARs Extracts from TB

The compositions of total ARs and individual homologues in triticale bran are shown in Table 2. The total amount of ARs found in triticale bran was 143.29 mg/100 g of which saturated ARs make up 82.0% and homologue C21:0 was the most prevalent with a concentration of 39.76 mg/100 g.

**Table 2:** Total alkylresorcinol (AR) content and composition of homologues (mg/100 g) in extract from triticale bran (TB) determined by HPLC.

AR Homologue (mg/100 g)	
Saturated	
5-n-heptadecylresorcinol C 15:0	1.06 ± 0.2
5-n-heptadecylresorcinol C 17:0	12.44 ± 0.5
5-n-nonadecanylresorcinol C 19:0	28.08 ± 0.3
5-n-heneicosylresorcinol C 21:0	39.76 ± 0.3
5-n-tricosylresorcinol C 23:0	20.33 ± 0.2
5-n-pentacosylresorcinol C 25:0	15.86 ± 0.1
Unsaturated	12.12 ± 0.2
Unknown	13.61 ± 0.6
Total	143.29 ± 0.3

Analyses were performed in triplicate and the results were expressed as mean ± SD.

### 3.2. Weight and Intake Parameters

**Figure 1:**
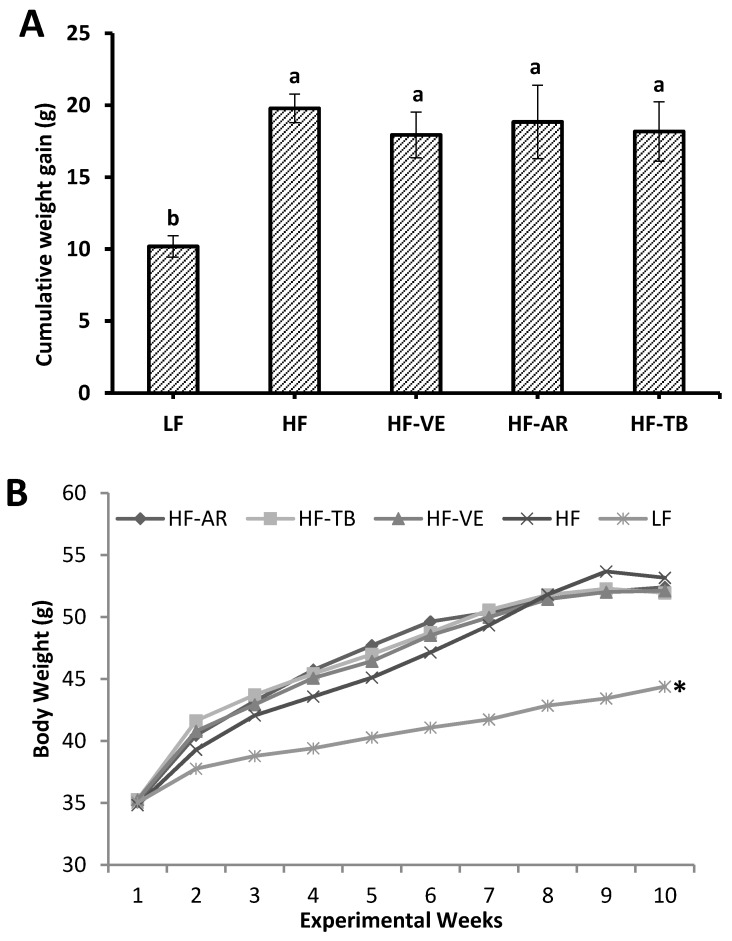
Cumulative weight gain (**A**) and body weight after 10 experimental weeks (**B**). LF: group fed standard low-fat diet; HF: group fed high-fat diet; HF-VE: group fed HF diet with 0.5% VE added; HF-AR: group fed HF diet with 0.5% ARs extract added; HF-TB: group fed HF diet with 10% TB. Bars represent mean ± SE. Data were statistically analyzed using one-way ANOVA and DMRT where statistical differences (*p* < 0.05) are represented by different letters and * indicates statistical difference from HF group.

**Figure 2:**
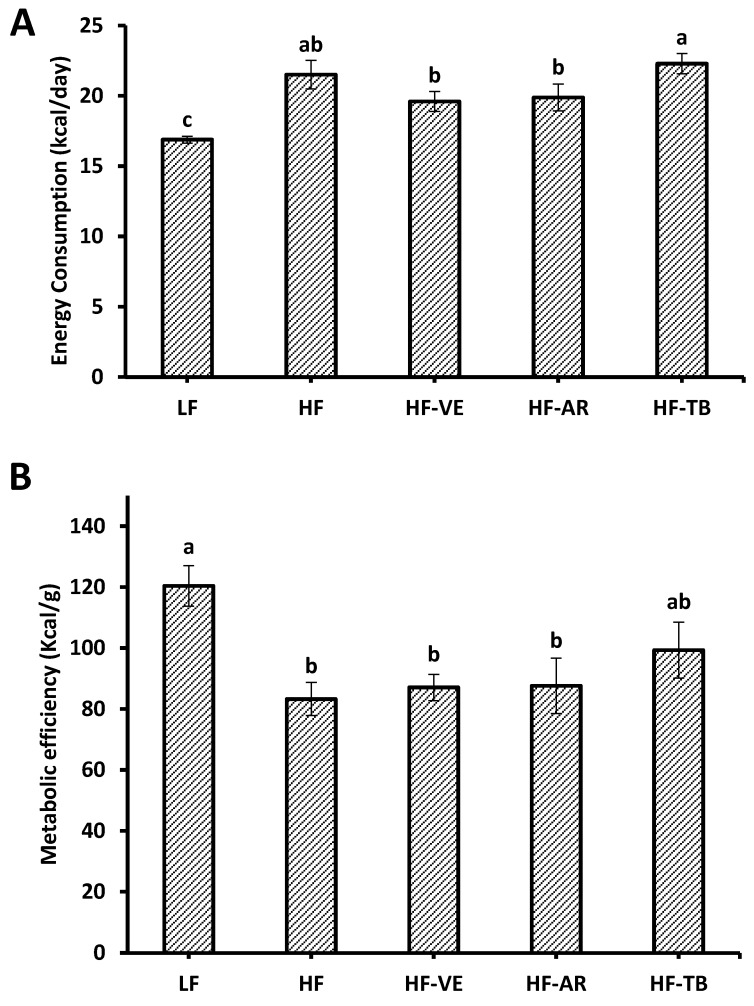
Energy consumption (**A**) and metabolic efficiency after 10 experimental weeks (**B**). LF: group fed standard low fat diet; HF: group fed high-fat diet; HF-VE: group fed HF diet with 0.5% VE added; HF-AR: group fed HF diet with 0.5% ARs extract added; HF-TB: group fed HF diet with 10% TB. Bars represent mean ± SE. Data were statistically analyzed using one-way ANOVA and DMRT where statistical differences (*p* < 0.05) are represented by different letters.

During the two-week baseline period in which all five groups were fed the same chow diet, groups showed similar means (*p* > 0.05) in weight (33.6–34.4 g), daily weight gain (0.15–0.22 g/day), and energy intake (17.3–18.4 Kcal/day). After the 10-week treatment period, animals fed high-fat diets were 21% heavier and exhibited a higher mean of daily weight gain (*p* < 0.05) compared to the standard control group LF. No statistical differences were found in cumulative weight gain or body weight among the HF treatment groups (HF-AR, HF-TB, HF-VE, and HF), as seen in Figure 1. Comparable results were found for the difference in mean energy intake (*p* < 0.05) between control groups HF and LF. On the other hand, the mean energy intake of groups HF and HF-TB was found to be significantly higher than that of HF-AR and HF-VE groups (Figure 2A). To estimate differences in the ability of ingested energy to be metabolized [23], metabolic efficiency (ME) across the 10-week treatment period was calculated as follows:

ME = energy intake/body weight gain.

While a statistically significant difference cannot be seen, the HF-TB group exhibits a greater mean metabolic efficiency of 99 Kcal/g of body weight 227 when compared to the 83 Kcal/g for control HF animals (Figure 2B). Overall, the standard control group LF demonstrated the lowest mean values in weight (44.3 g), daily weight gain (0.17 g), energy intake (16.8 Kcal), and greatest metabolic efficiency (120.4 Kcal/g).

### 3.3. Glucose Tolerance and Fasting Blood Glucose

**Figure 3:**
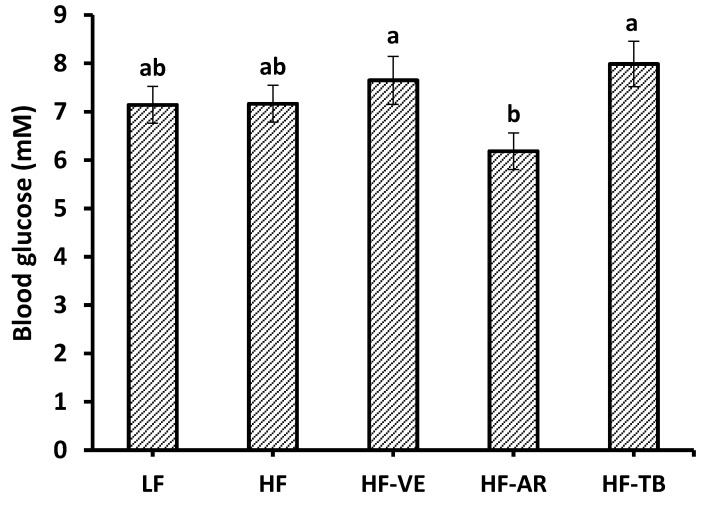
Fasting blood glucose levels after 10 experimental weeks. LF: group fed standard low fat diet; HF: group fed high-fat diet; HF-VE: group fed HF diet with 0.5% VE added; HF-AR: group fed HF diet with 0.5% ARs extract added; HF-TB: group fed HF diet with 10% TB. Bars represent mean ± SE. Data were statistically analyzed using one-way ANOVA and DMRT where statistical differences (*p* < 0.05) are represented by different letters.

**Figure 4:**
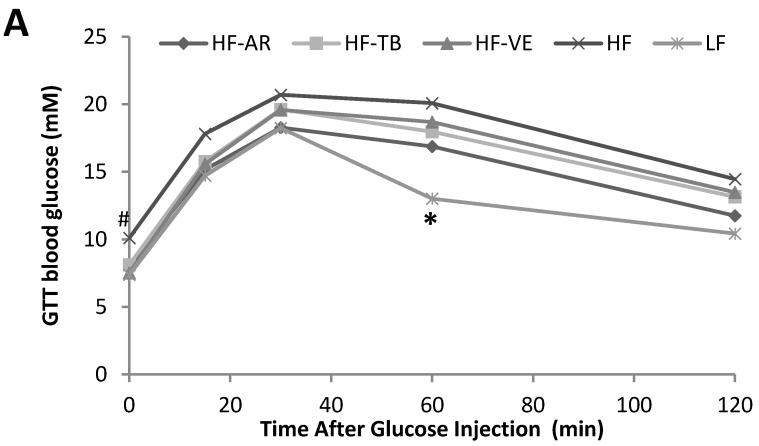

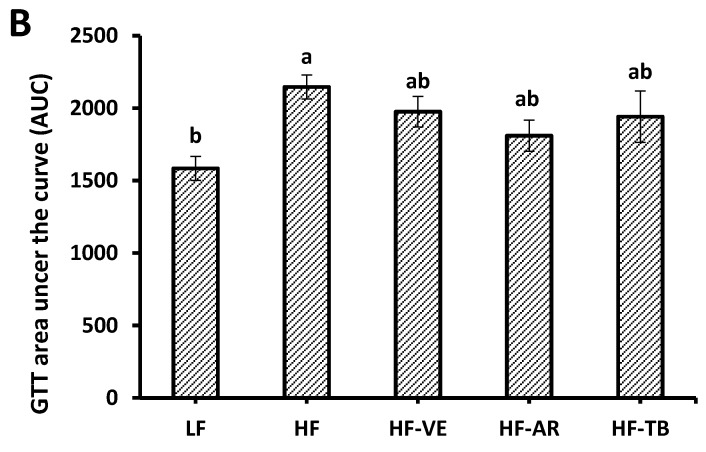
Results of glucose tolerance test (GTT) mean blood glucose levels at 0, 30, 60, 90 and 120 min after intraperitoneal injection of glucose solution (**A**), and mean area under the curve of GTT graph (**B**). LF: group fed standard low fat diet; HF: group fed high-fat diet; HF-VE: group fed HF diet with 0.5% VE added; HF-AR: group fed HF diet with 0.5% ARs extract added; HF-TB: group fed HF diet with 10% TB. Bars represent mean ± SE. Data were statistically analyzed using one-way ANOVA and DMRT where statistical differences (*p* < 0.05) are represented by different letters. # indicates statistical difference from the control group and * indicates statistical difference from HF group.

Although differencs were not statistically significant, lower fasting blood glucose levels of animals on the HF-AR diet (6.1 mM) than the remaining HF treatment groups (7.2–8.0 mM) and control LF group (7.1 mM) are apparent in Figure 3. The results of the GTT seen in Figure 4A indicated no significant difference among all five groups at 15, 30, and 120 min. At 0 min blood glucose levels were significantly higher in the HF group, with no marked differences found between the remaining groups. After 60 min, control LF mice exhibited a significantly lower drop in blood glucose than the HF control and treatment groups, with the exception of HF-AR. At each of the time points throughout the GTT, HF-AR mice had the lowest blood glucose levels among the HF groups. Results of the GTT at different time points were used to calculate the area under the curve (AUC) in the glucose *versus* time graph. Based on results of AUC (Figure 4B), mice fed HF alone had a significantly higher mean AUC (2146.5) than that of mice fed the standard LF (1585.1). Among the HF groups, HF-AR had the lowest AUC (1810.8) followed by HF-TB (1941.5), HF-VE (1976.0), and HF.

### 3.4. Body Composition

The values obtained for carcass analysis at the end of the experimental period are summarized in Table 3. No statistical difference was found in % fat amongst the HF groups irrespective of treatment received. However, HF-TB had the least % fat (24.1%) and statistically highest % lean muscle (72.9%) when compared with the remaining HF groups. These results depict a similar trend to that of metabolic efficiency findings. These findings suggest that incorporation of 0.5% AR extract or 0.5% vitamin E in a 60% high-fat diet does not prevent fat accumulation; however, incorporation of 10% TB may improve metabolic efficiency and promote an increase in % lean muscle.

**Table 3:** Body composition of mice carcasses.

Treatment ^1^	Weight (g)	Fat (%)	Lean Muscle (%)
LF	36.60 ± 1.35 ^b^	18.02 ± 1.00 ^b^	75.90 ± 0.89 ^a^
HF	46.66 ± 1.06 ^a^	27.00 ± 1.03 ^a^	69.89 ± 1.35 ^c^
HF-VE	45.17 ± 2.18 ^a^	26.09 ± 0.63 ^a^	70.87 ± 0.62 ^bc^
HF-AR	45.83 ± 3.15 ^a^	28.10 ± 1.92 ^a^	67.79 ± 1.89 ^c^
HF-TB	45.06 ± 2.32 ^a^	24.14 ± 1.35 ^a^	72.87 ± 1.43 ^ab^

Body composition data obtained by EchoMRI4in1TM and expressed as % of decapitated mouse carcass body weight. ^1^ LF: group fed standard low-fat diet; HF: group fed high-fat diet; HF-VE: group fed HF diet with 0.5% VE added; HF-AR: group fed HF diet with 0.5% ARs extract added; HF-TB: group fed HF diet with 10% TB. Data are presented as mean ± SE. Data were statistically analyzed using one-way ANOVA and DMRT where statistical differences among groups (*p* < 0.05) are represented by different letters.

### 3.5. Oxygen Radical Absorbance Capacity (ORAC)

**Figure 5:**
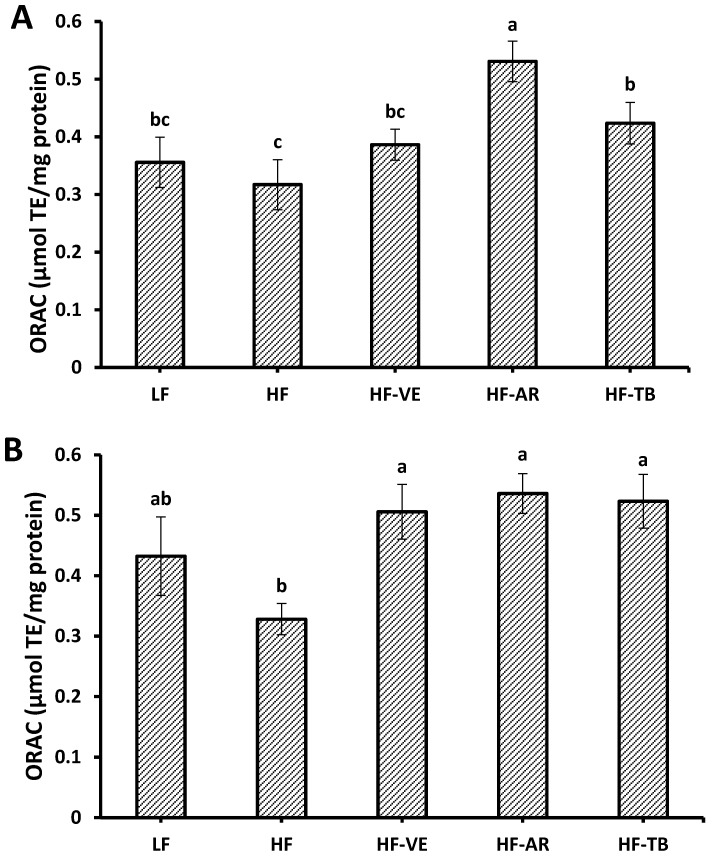
Total antioxidant activity in liver (**A**) and heart tissue of mice as evaluated by the ORAC method (**B**). LF: group fed standard low-fat diet; HF: group fed high-fat diet; HF-VE: group fed HF diet with 0.5% VE added; HF-AR: group fed HF diet with 0.5% ARs extract added; HF-TB: group fed HF diet with 10% TB. Bars represent mean ± SE. Data were statistically analyzed using one-way ANOVA and DMRT where statistical differences among groups (*p* < 0.05) are represented by different letters.

The ORAC assay showed that mice fed a high-fat diet demonstrated poor antioxidant protection of the fluorescent probe from peroxyl radicals generated by APPH. Liver and heart tissues of HF control mice were found to have the lowest ORAC values of 0.31 and 0.33 µmol TE/g of protein, respectively, among all the groups (Figure 5). Supplementation of the high-fat diet with 0.5% AR extract significantly improved antioxidant status (*p* < 0.05), as evidenced by much higher mean ORAC values of 0.53 and 0.54 µmol TE/g of protein, respectively. Addition of 0.5% VE and 10% TB also showed marked improvements of 25%–35% in the liver and 55%–65% in the heart compared to the tissues of non-supplemented HF mice.

### 3.6. Superoxide Dismutase (SOD) Activity

**Figure 6:**
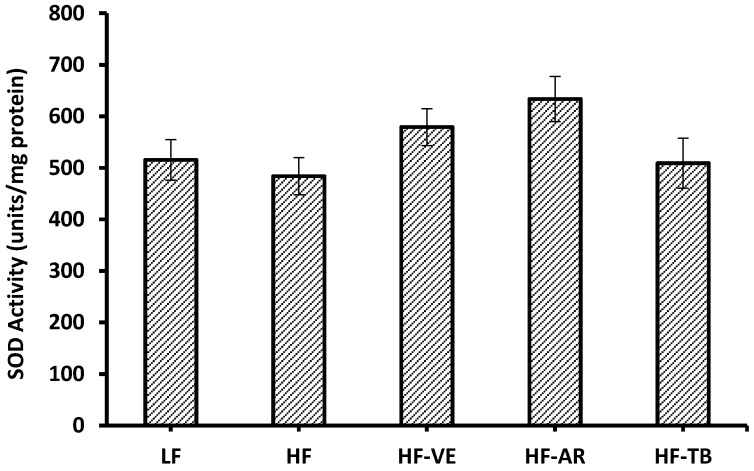
Superoxide dismutase activity in liver tissue of mice. LF: group fed standard low-fat diet; HF: group fed high-fat diet; HF-VE: group fed HF diet with 0.5% VE added; HF-AR: group fed HF diet with 0.5% ARs extract added; HF-TB: group fed HF diet with 10% TB. Bars represent mean ± SE. Data were statistically analyzed using one-way ANOVA and DMRT where no statistical differences among groups were found (*p* > 0.05).

Although ARs seemed to improve SOD activity levels by 30% when compared to control HF liver tissues, this difference was not statistically significant (*p* > 0.05). The same was true for the remainder of the groups such that the activity of the SOD enzyme increased by approximately 20% and 5% for VE and TB supplemented mice, respectively (Figure 6). In order to quantify units of SOD per mg of protein, differences in % inhibition of NADH oxidation must be observed at varying concentrations of tissue assayed. For this reason, no quantifiable differences were found for the SOD activity in mice heart tissues.

### 3.7. Reduced Glutathione (GSH) Content

**Figure 7:**
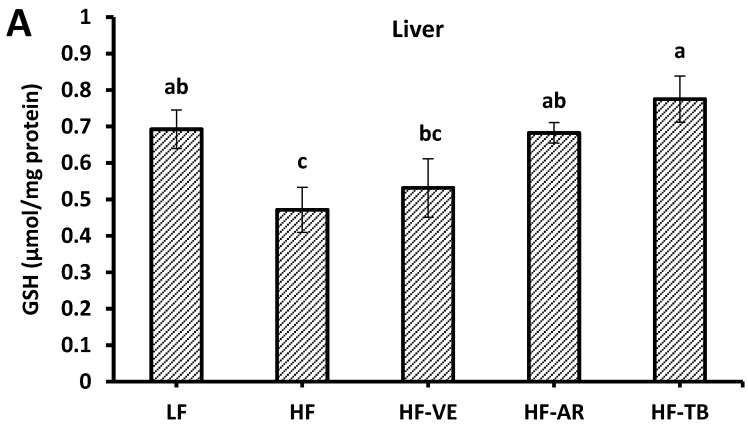

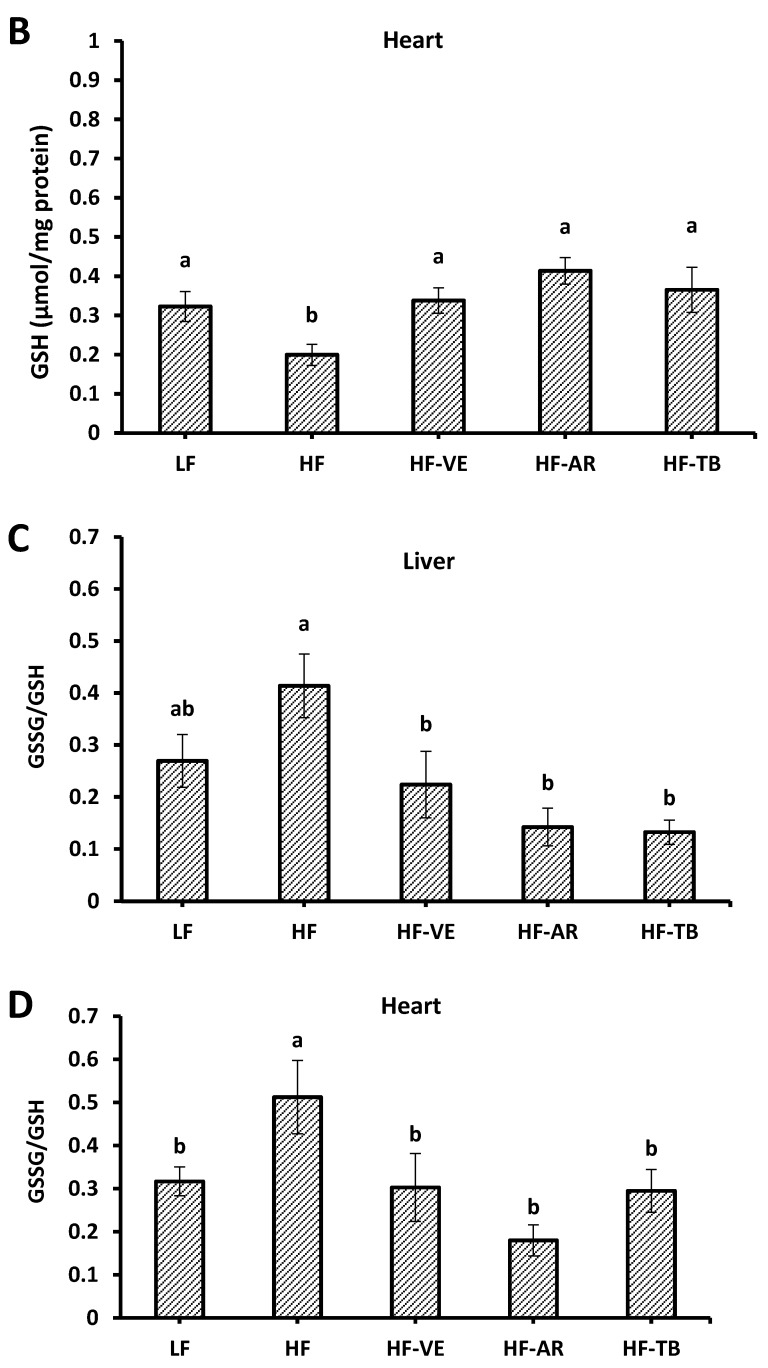
Reduced glutathione levels (**A** & **C**) and ratio of oxidized glutathione to reduced glutathione (GSSG/GSH) in mice liver and heart tissues (**B** & **D**). LF: group fed standard low-fat diet; HF: group fed high-fat diet; HF-VE: group fed HF diet with 0.5% VE added; HF-AR: group fed HF diet with 0.5% ARs extract added; HF-TB: group fed HF diet with 10% TB. Bars represent mean ± SE. Data were statistically analyzed using one-way ANOVA and DMRT where statistical differences among groups (*p* < 0.05) are represented by different letters.

Liver and heart tissues of the control HF group exhibited decreased levels of reduced GSH (0.47 µM and 0.20 GSH/mg of protein) and increased ratios of GSSG/GSH (0.41 and 0.51), respectively, in comparison to control LF mice (0.69 and 0.32 µM GSH/mg of protein; 0.27 and 0.32 GSSG/GSH). These results were expected since a high-fat diet is often correlated with increasing levels of ROS, leading to oxidative stress [24]. Overall, liver and heart tissues from HF-TB and HF-AR mice had significantly higher GSH levels and significantly lower ratios of GSSG/GSH (*p* > 0.05) in comparison to mice fed the high-fat diet alone (Figure 7).

## 4. Discussion

### 4.1. Weight and Intake Parameters

An increasing number of studies have reported the beneficial effects of phenolic compounds and bioactives in cereal grains on obesity. Phenolics such as anthocyanins, rutin, gallic acid, ferulic acid, genestein, and tea catechins can induce apoptosis of adipocytes, inhibit trigycleride accumulation, or regulate plasma lipids and cholesterol levels; these are some of the mechanisms that have been identified in which phenolics can act against the prevalent disease [25,26]. In this study, neither AR nor VE supplementation resulted in significant changes in body weight, composition, or metabolic efficiency. Ross *et al.* [27] found coinciding results in which rats fed a basal diet supplemented with 4 g AR/kg feed for fourweeks showed no effects on final body or organ weights. Nonetheless, the higher energy intake yet greater metabolic efficiency found for animals fed HF-TB compared to HF alone indicates that consumption of TB did attenuate weight gain and enhance metabolism in some respect, which is in agreement with existing literature [28,29].

### 4.2. Glucose Tolerance and Fasting Blood Glucose

An impaired glucose tolerance test and persistently high FBG levels are indicators of insulin resistance and hyperglycemia, respectively; these conditions are often linked to obesity and diabetes. Insulin resistance is the failure of cells to respond to insulin hormone signaling, leading to reduced glucose uptake and hyperglycemia [23,30]. Although not statistically significant (*p* = 0.06), the addition of ARs did improve glucose tolerance, as evidenced by a lower mean area under the curve. Additionally, fasting blood glucose levels were significantly lower in mice fed HF-AR rather than HF alone. A recent study by Magnusdottir *et al.* [31] discovered an association between the plasma C17:0/C21:0 AR homolog ratio and increased insulin sensitivity in subjects with metabolic syndrome fed a diet rich in whole grain rye. In examining the intestinal loops of anesthetized dogs administered a solution of alkyresorcinol and insulin, Sealock *et al.* [32] found that alkylresorcinols and related compounds promoted the absorption of insulin from the gastrointestinal tract and into the circulating bloodstream, thereby significantly dropping blood glucose levels. Results of these studies suggest that ARs perhaps interact with insulin in a synergistic manner, potentially enhancing the functionality of insulin and its effectiveness in blood glucose clearance. Although further investigation is necessary to confirm this prospect and understand its mechanism of action, ARs show promising results in terms of their potential to ameliorate the glucose intolerance and insulin sensitivity commonly associated with obesity and diabetes [17].

### 4.3. Oxygen Radical Absorbance Capacity (ORAC)

The ORAC assay best represents antioxidant reactions within biological systems by providing a controllable source of peroxyl radicals that react with a florescent probe, resulting in a non-fluorescent product; antioxidant capacity is quantified by comparison of net AUC (relative fluorescence units *vs.* time) to that of a known antioxidant, trolox [33]. The significantly higher ORAC levels in the liver and heart tissues of mice fed a high-fat diet with 0.5% ARs confirms the literature results reporting the *in vitro* antioxidant potential of these dynamic phenolic lipids [11,16,34,35,36,37]. It is speculated that the amphiphilic nature of ARs carries out their antioxidant actions in a similar manner to that of tocopherols by easily incorporating in cell lipid bilayers and providing protection against lipid peroxidation by hydrogen atom transfer from hydroxyl groups of the phenol ring to peroxyl radicals, thereby forming stable products [10].

### 4.4. Superoxide Dismutase (SOD) Activity

Superoxide dismutase is an enzyme that catalyzes the dismutation of superoxide (O_2_**^.^** ^−^) radicals into less reactive forms, hydrogen peroxide and oxygen. It is proposed that the superoxide anion can initiate and terminate lipid peroxidation, thus SOD concentrations in mammalian cells are not easily susceptible to change as enough SOD is expressed to predominantly suppress ROS imbalances linked to superoxide [38]. Also, it is known that SOD is a strong antioxidant that out-competes the reactions of O_2_**^.^** ^−^ to protect the cell from its toxicity [39]. The supplementation of 0.5% antioxidants (ARs or VE) with a high-fat diet did not significantly enhance SOD activity. However, the expected negative impact on SOD activity associated with increased levels of metabolic stressors such as ROS due to a high-fat diet [3] were also not statistically pronounced. The lack of pronounced effects on SOD activity, whether negative or positive, is potentially due to the strength and self-sufficient nature of the SOD enzyme in successfully suppressing superoxide linked chain reactions. Thus, is it theorized that for significant changes in SOD activity to be apparent, the initiating source of oxidative stress must be substantially stronger and present for a more prolonged period of time than that experienced by the high-fat fed mice in this study. In the future, other antioxidant enzymes such as glutathione peroxidase and catalase may be a more reliable measure of antioxidant effects in less extreme conditions of oxidative stress.

### 4.5. Reduced Glutathione (GSH) Contents

A chief contributor to the biological defense system against oxidative damage is the antioxidant molecule glutathione, which is naturally synthesized in the body. In its reduced form, it directly interacts with reactive oxygen and nitrogen species or acts as a cofactor for enzymes. In healthy cells, glutathione is mainly in the reduced form and a rising ratio of oxidized to reduced glutathione (GSSG/GSH) is a significant marker of oxidative stress [40]. The significant increase in GSH levels and decrease in the ratio of GSSG/GSH in HF-AR liver and heart tissues suggest that a diet supplemented with ARs may play a role in the recycling of GSSG back to its reduced form as GSH. Although the mechanism of action requires further investigation, it would be presumed that ARs help increase GSH levels by direct reduction of GSSG through hydrogen atom transfer/donation or by free radical quenching, which indirectly helps maintain higher GSH levels. Since GSH has multiple roles in the endogenous antioxidant defense system, it is likely that GSH cooperates with SOD in the removal of free radicals [39]. This may explain the significant effects demonstrated by ARs on GSH, yet less pronounced or undetectable effects on SOD activity in the liver and heart, respectively, as ARs may only impact SOD activity indirectly through GSH.

## 5. Conclusions

Cereal grains are rich in phenolics, predominantly in the bran fraction, which also contains an abundance of other bioactive components that have been shown to protect against chronic diseases including obesity and diabetes [41,42]. Although ARs did not impact weight gain and body composition, results were promising in their ability to improve glucose tolerance and fasting blood glucose levels. Additionally, ARs proved to have antioxidant potential *in vivo* through their enhanced effects on oxidative stress markers, results that confirmed literature findings for the antioxidant capacity of ARs demonstrated *in vitro*. Alkylresorcinols are known to be weaker antioxidants than tocopherols based on *in vitro* studies [11]; however, results of this study demonstrate that several other physiological factors are involved *in vivo* as ARs had an overall greater effect than VE in improving oxidative stress as well as glucose tolerance. It has also been shown that AR chain length has an effect on their *in vivo* elimination kinetics such that the half-life of ARs in rats was positively correlated with alkyl chain length [43]. However, Korycinska *et al.* [11] found that chain length did not significantly impact their antioxidant status. Thus, future studies aim to measure and compare the effects of individual AR homologs *in vivo*. Also, the complex nature of the mechanisms involved in the body’s antioxidant system requires further exploration to better understand the effects of antioxidant therapy and apply these findings to preventative measures against oxidative stress and chronic diseases.
